# Can a Brief Relaxation Exercise Modulate Placebo or Nocebo Effects in a Visceral Pain Model?

**DOI:** 10.3389/fpsyt.2019.00144

**Published:** 2019-03-21

**Authors:** Sigrid Elsenbruch, Till Roderigo, Paul Enck, Sven Benson

**Affiliations:** ^1^Institute of Medical Psychology and Behavioral Immunobiology, University Hospital Essen, Essen, Germany; ^2^Department of Internal Medicine VI, University Hospital Tuebingen, Tuebingen, Germany

**Keywords:** placebo effect, nocebo effect, expectation, visceral pain, pain unpleasantness, stress, relaxation

## Abstract

Translational research aiming to elucidate mediators and moderators of placebo and nocebo effects is highly relevant. This experimental study tested effects of a brief progressive muscle relaxation (PMR) exercise, designed to alter psychobiological stress parameters, on the magnitude of placebo and nocebo effects in a standardized psychosocial treatment context. In 120 healthy volunteers (60 men, 60 women), pain expectation, pain intensity, and pain unpleasantness in response to individually-calibrated rectal distensions were measured with visual analog scales during a baseline. Participants were then randomized to exercise PMR (relaxation group: *N* = 60) or a simple task (control group: *N* = 60), prior to receiving positive (placebo), negative (nocebo) or neutral suggestions regarding an intravenous administration that was in reality saline in all groups. Identical distensions were repeated (test). State anxiety, salivary cortisol, heart rate, and blood pressure were assessed repeatedly. Data were analyzed using analysis of covariance, planned Bonferroni-corrected group comparisons, as well as exploratory correlational and mediation analyses. Treatment suggestions induced group-specific changes in pain expectation, with significantly *reduced* expectation in placebo and *increased* expectation in nocebo groups. PMR had no discernable effect on pain expectation, state anxiety or cortisol, but led to significantly lower heart rate and systolic blood pressure. Relaxation significantly interacted with positive treatment suggestions, which only induced placebo analgesia in relaxed participants. No effects of negative suggestions were found in planned group comparisons, irrespective of relaxation. Exploratory correlation and mediation analyses revealed that pain expectation was a mediator to explain the association between treatment suggestions and pain-related outcomes. Clearly, visceral pain modulation is complex and involves many cognitive, emotional, and possibly neurobiological factors that remain to be fully understood. Our findings suggest that a brief relaxation exercise may facilitate the induction of placebo analgesia by positive when compared to neutral treatment suggestions. They underscore the contribution of relaxation and stress as psychobiological states within the psychosocial treatment context—factors which clearly deserve more attention in translational studies aiming to maximize positive expectancy effects in clinical settings.

## Introduction

Although placebo research spans many medical disciplines, the pain field continues to drive conceptual, mechanistic, and clinical advances in placebo knowledge, providing fruitful opportunities of forward- and backward translation. Placebo analgesia constitutes one of the most fascinating and impressive examples of such translational research. Laboratory and preclinical studies in healthy populations and in patients with chronic pain conditions have elucidated the psychological and neurobiological mechanisms underlying placebo and nocebo effects (1, 2). The clinical potential offered by a transfer of this knowledge into treatment settings has been recognized within the pain field (3, 4) and beyond (5). This is underscored by trials supporting the efficacy of placebo interventions in patients with chronic low back pain (6, 7) and chronic visceral pain (8, 9). Facilitating placebo while minimizing nocebo effects may contribute to refining treatment approaches to provide patients with improved and more personalized patient care (10, 11). Toward this end, translational research aiming to optimize the efficacy of placebo interventions is highly relevant. In the context of chronic visceral pain and related gastrointestinal symptoms, the potential of placebo knowledge has been recognized but is far from fulfilled (12–14).

Various aspects of the psychosocial treatment context, including the setting (15), nature of the intervention, as well as the quality and quantity of patient-provider interactions (9, 16, 17), shape treatment expectations and thereby the presence and magnitude of placebo effects. Optimizing the psychosocial treatment context has the potential to improve the efficacy of placebo treatment, and to maximize the benefits of placebo-elements that are an inherent part of therapeutic interventions, including pharmacological treatments (4, 18). Interestingly, two laboratory studies in healthy volunteers support the idea that placebo analgesia can be enhanced with specific pharmacological interventions, i.e., the administration of vasopressin and oxytocin, respectively (19, 20). Whether behavioral approaches that target stress-related psychobiological factors are capable of facilitating placebo analgesia has not been tested. Herein, we explore for the first time the modulatory effects of a brief behavioral intervention, i.e., progressive muscle relaxation (PMR), on placebo and nocebo effects in a clinically-relevant model of visceral pain. The rationale was inspired by evidence supporting that *enhanced* stress [e.g., increased state anxiety (21–24), subjective stress levels (25–28), experimentally-induced fear (29), acute psychosocial stress (30)] moderates placebo and/or nocebo effects. As part of a larger experimental study (30), we herein implemented PMR aiming to test effects of *reduced* stress-related psychobiological factors on the magnitude of placebo and nocebo effects induced by treatment suggestions. Building on our earlier experimental studies on placebo/nocebo effects in the context of visceral pain (22, 30–33), we specifically aimed to test whether a brief relaxation exercise, carried out immediately prior to the delivery of deceptive positive (placebo), deceptive negative (nocebo), or truthful neutral (control) treatment suggestions, can facilitate placebo analgesia or reduce nocebo hyperalgesia in an established and clinically-relevant model of visceral pain in healthy volunteers. To explore if the effects of relaxation or treatment suggestions on outcomes were mediated by stress markers or expectations, we conducted correlational and mediation analyses.

## Materials and Methods

### Participants

Healthy adults were recruited by local advertisements seeking volunteers for an experimental study on the modulation of visceral pain perception. We herein report on a total of *N* = 120 healthy volunteers (60 men, 60 women) who were randomized to a brief relaxation exercise or a control task on the experimental study day just prior to undergoing an established placebo/nocebo paradigm (see below, study design). Note that this study was conducted as part of a larger trial which also included an additional *N* = 60 volunteers who were randomized to a psychosocial stress protocol (data on the psychosocial stress and control groups have been reported in Roderigo et al. (30). Recruitment and screening procedures were accomplished with a total of *N* = 219 participants originally interested in the study. Reasons for non-participation were lack of interest, exclusion based on criteria specified below, and a high pain threshold that was above the herein applied safety cut-off for distensions at 55 mmHg. The study was conducted at Essen University Hospital with data collection between January 2015 and June 2016. The study protocol was approved by the local ethics committee (protocol number 13-5565-BO, approval date: August 28, 2013). All volunteers gave informed written consent in accordance with the Declaration of Helsinki and were paid for their participation.

Exclusion criteria included age <18 or >65 years, a body mass index (BMI) <18 or >30, any known medical or psychological conditions, current medication use (except thyroid medication, occasional over-the-counter drugs for minor allergies, benign headaches, etc.), current anxiety or depression symptoms above the published cut-off values on the Hospital Anxiety and Depression Scale (HADS) (34), current gastrointestinal (GI) symptoms suggestive of an undiagnosed GI condition (35), peri-anal tissue damage (e.g., painful hemorrhoids or fissures which may interfere with rectal balloon placement), and prior participation in any of our previous placebo studies. In an effort to reduce possible variability related to fluctuations of hormones across the female menstrual cycle, only women on hormonal contraceptives were recruited. All participants completed a comprehensive questionnaire battery, as detailed in Roderigo et al. (30). We herein characterized groups using the HADS (34) for symptoms of anxiety and depression, the trait version of the STAI (36) for trait anxiety, the TICS (screening scale) (37) for chronic perceived stress, and sum scores from a gastrointestinal (GI) symptom questionnaire (35) to assess frequency and severity of common upper and lower GI symptoms. Note that previous experience with any type of relaxation technique, including progressive muscle relaxation (PMR) was not an inclusion or exclusion criterion, however, it was required that volunteers were willing to complete a home-based PMR training program as part of the study, as detailed below.

### Study Design and Procedures

During a 4-week period preceding the experimental study day, volunteers were instructed to complete a home-based, standardized training program in progressive muscle relaxation (PMR). This was done to achieve a large enough sample of individuals capable of completing a short relaxation exercise on the day of the study. In order to achieve proper blinding and randomization, all 180 participants underwent the training program. To do so, we selected a commercially available training manual that consisted of an illustrated book with an audio CD that contained guided training sessions. Note that the same audio-guided training CD was used by participants randomized to the brief relaxation group on the study day. Every volunteer—irrespective of possible prior experience with the PMR or other relaxation techniques—was instructed to start the training in the first week with two sessions of a long program that lasted ~40 min. Thereafter, participants could choose between the long version and a shorter 15-min. version in the remaining training weeks, but were required to practice at least twice per week. Participants recorded their practice in a training log, and at the end of the week (i.e., on Sundays) completed a standardized questionnaire assessing the number of training sessions (N), training duration (in minutes), perceived training efficacy (7-point Likert-scale ranging from “training worked not at all” to “training worked perfectly”), psychological distress (7-point Likert-scale ranging from “felt completely relaxed” to “felt extremely distressed”) and various bodily symptoms (not reported here) for the past week. Together with each weekly questionnaire, participants collected morning saliva samples for analysis of the cortisol awakening response (CAR). In case of non-compliance (i.e., on average <2 training sessions per week) participants were encouraged to continue practicing for up to two additional weeks before the study day was scheduled. Note that questionnaire data and CAR were not acquired to verify the efficacy of PMR training (which is impossible given the absence of a control group that did not undergo training) but rather to provide sample characteristics for comparisons of groups that on the study day were randomized to brief relaxation exercise vs. a control task.

On the experimental study day, rectal sensory and pain thresholds were initially determined with a pressure-controlled barostat system (modified ISOBAR 3 device, G & J Electronics, Ontario, Canada), using well-established methodology [e.g., (22, 30–33, 38). During a BASELINE, each participant received a series of painful rectal distensions titrated individually to rectal threshold (6 distensions, duration each 30 s; pauses in-between 30 s). Participants were then randomized to relaxation (practice relaxation using the 15-min. audio-CD program, *N* = 60) or control intervention (engage in an easy cognitive activity, e.g., crosswords, reading a magazine, *N* = 60) while stratifying for sex. Immediately afterwards, participants were randomized to positive (placebo), negative (nocebo), or neutral treatment suggestions (details on suggestions below). This resulted in a total of 2 (relaxation, control) x 3 (positive, negative, neutral suggestions) experimental groups consisting of *N* = 20 participants per group. The series of rectal distensions using the same individualized pressures as during BASELINE was then repeated (TEST).

### Treatment Suggestions and Blinding

We herein implemented previously used methodology to induce placebo and nocebo effects in this visceral pain model [e.g., (30, 33); for recent discussions of methodology aspects, see (13, 14)]. In this paradigm, deceptive or truthful treatment suggestions are delivered in combination with an i.v. administration that in reality contains saline. In placebo groups, volunteers receive positive treatment suggestions regarding pain relief induced by a spasmolytic drug (i.e., Butylscopolaminiumbromid). In nocebo groups, negative suggestions regarding increased pain sensitivity due to administration of an opioid antagonist (i.e., Naloxone) are delivered. In control groups, truthful information about saline are provided. These control groups (herein referred to as “neutral” groups to distinguish from the relaxation vs. control intervention group terminology) are an essential part of the study design as they allow a differentiation and separate analyses of placebo and nocebo effects, respectively, as well as controlling for effects of time (e.g., habituation), etc.

In order to achieve proper blinding and a randomization to treatment suggestions on the study day, all volunteers received deceptive information about all possible drug treatments during recruitment and informed consent, including detailed information about typical clinical uses, pharmacodynamics, and possible side effects. Blinding of the study team interacting with volunteers on the study day was accomplished as follows: The clinical psychologist responsible for recruitment and conducting the study protocol (relaxation, control) was blinded to subsequent treatment information, the physician who delivered treatment information was blinded to prior relaxation vs. control intervention, the female study nurse was fully blinded throughout the study day.

### Pain-Related Measures

Primary outcome measures were overall perceived visceral pain intensity and pain unpleasantness, quantified with visual analogs scales (VAS, 0−100 mm, ends defined as none—very much). In addition, expected pain intensity was quantified with a VAS (0−100 mm, ends defined as none—very much) prior to BASELINE and TEST, respectively.

### Additional Measures

State anxiety (STAI-S), salivary cortisol concentrations (see below), heart rate (Task Force Monitor, CNSystems Medizintechnik AG, Graz, Austria), and blood pressure were assessed repeatedly and are herein presented for a baseline (prior to first randomization to relaxation vs. control intervention), after treatment suggestions, and after the TEST series of distensions. Note that we chose not to additionally assess these stress-related measures in-between the intervention and delivery of treatment suggestions given concerns that this may disrupt or interfere with effects of relaxation on the subsequent experimental procedures.

Saliva samples were collected using Salivettes (Sarstedt, Nümbrecht, Germany). To assess the cortisol awakening response (CAR) during the 4-week home PMR training period, participants collected samples once per week immediately after awakening and 30, 45, 45, and 60 min. afterwards and stored the samples in their freezers until bringing them to the laboratory on the study day. All saliva samples, including all samples collected on the study day, were centrifuged (2,000 rpm, 2 min, 4°C) and stored at −20°C. Salivary cortisol concentrations were measured using a commercially available enzyme-linked immunosorbent assay (ELISA; IBL International, Hamburg, Germany) according to the manufacturer's protocol. Intra- and interassay variances were 4.8 and 5.9%, respectively. The detection limit was 0.138 nmol/l. The CAR was calculated as area under the curve (AUC) with respect to increase, which corrects for baseline levels, according to published recommendations (39).

### Statistical Analyses

All statistical analyses were conducted using SPSS version 22.0 (IBM Corporation, Armonk, NY). Power analysis using G-Power (http://www.gpower.hhu.de/) indicated that a total sample size of *N* = 120 has a sufficient statistical power of 1-β = 0.96 to detect large effects (f = 0.40, α = 0.05) for ANOVA interaction effects. The groups were characterized and compared with respect to sociodemographic, psychological, and clinical characteristics using Chi-Square Tests, *t*-tests, or Mann-Whitney-*U*-tests where appropriate.

Effects of the relaxation vs. control on stress markers were tested with repeated measures analysis of covariance (ANCOVA) with time as repeated factor and two between factors, namely intervention (relaxation, control) and treatment suggestions (positive, negative, neutral). Note that the factor “treatment suggestions” was included as a group factor in this analysis to test for possible interactions between the intervention and treatment suggestions on stress markers. *Post hoc* tests were conducted as Bonferroni-corrected ANCOVA (for comparisons between groups) or Bonferroni-corrected paired *t*-tests (for changes across time points within one group).

To address effects of relaxation and treatment suggestions on changes in pain expectation, pain intensity, and pain unpleasantness from BASELINE to TEST, repeated measures ANCOVAs were computed with the repeated factor time and two group factors (intervention; treatment suggestions). Bonferroni-corrected planned comparisons of pre-specified group means were accomplished with univariate ANCOVAs testing differences between positive and neutral treatment suggestion groups (for placebo effects) and between negative and neutral treatment suggestion groups (for nocebo effects). In all ANCOVAs, Greenhouse-Geisser correction was applied if the sphericity assumption was violated (based on results of Mauchly test), and HADS anxiety scores were included as a covariate, given a small but significant group difference between the relaxation and control groups (see results, Table 1).

**Table 1:** Sample characteristics and measures collected during training.

	**Relaxation group (*N* = 60)**	**Control group (*N* = 60)**	**Test statistic**	***P***
**SOCIODEMOGRAPHIC AND PSYCHOSOCIAL VARIABLES (QUESTIONNAIRE BATTERY)**				
Sex [Women (*N*): Men (*N*)]	30 : 30	30 : 30	–	–
Age, years	27.2 ± 0.9	26.4 ± 0.8	*t* = −0.7	0.51
Body mass index	23.5 ± 0.3	23.2 ± 0.4	*t* = −0.5	0.60
Education (≥ high school degree), % (*N*)	92 (55)	90 (54)	X^2^ = 0.1	0.75
Married or partner, % (*N*)	58 (35)	60 (36)	X^2^ = 0.1	0.77
Non-smoker, % *N*	78 (47)	88 (53)	X^2^ = 2.2	0.14
Gastrointestinal symptom sum score	3.1 ± 0.3	3.6 ± 0.4	*t* = 1.0	0.31
HADS depression symptoms	2.1 ± 0.3	2.1 ± 0.3	*t* = 0.2	0.87
HADS anxiety symptoms	3.9 ± 0.3	5.0 ± 0.4	*t* = 2.3	0.026
STAI Trait anxiety	35.0 ± 0.9	36.5 ± 1.2	*t* = 1.0	0.32
TICS Chronic stress	17.1 ± 1.0	18.5± 1.1	*t* = 0.8	0.35
**TRAINING PERIOD^a^ (DIARY)**				
Mean training time, minutes	75.6 ± 8.8	66.3 ± 5.9	*t* = −0.9	0.38
Mean training sessions, *N*	2.8 ± 0.2	2.5 ± 0.1	*t* = −1.3	0.20
Perceived training efficacy^b^	4.6 ± 0.1	4.6 ± 0.1	U = −0.1	0.91
Psychological distress^c^	4.0 ± 0.1	4.1 ± 0.1	U = −0.2	0.88
Cortisol awakening response (nmol/l)^d^	242.7 ± 65.0	263.1 ± 61.8	*t* = 0.22	0.82

*All data are shown as mean ± standard error of the mean, unless indicated otherwise. For all questionnaire references, see main text. HADS, Hospital Anxiety and Depression Scale; STAI, Spielberger State-Trait Anxiety Inventory (trait version); TICS, Trier Inventory for Chronic Stress (screening scale)*;

a *Mean values averaged over weekly diaries completed during the 4-wk training period. For detailed weekly results*.

b Perceived training efficacy during the last week, rated on a seven-point Likert-scale ranging from “training worked not at all” to “training worked perfectly.”

c Mean distress in past week rated on a seven-point Likert-scale ranging from “felt completely relaxed” to “felt extremely distressed.”

d *Cortisol awakening response measured once per week, calculated as area under the curve (AUC) with respect to increase which controls for baseline levels*.

To explore if the effects of relaxation or treatment suggestions on outcomes were mediated by stress markers or expectations, we conducted correlational and mediation analyses. Correlations were computed as Pearson's r. Mediation analyses were conducted using the PROCESS SPSS macro provided by A.F. Hayes (version 2.12.2, downloaded from http://www.processmacro.org/download.html). Bootstrapping with 10,000 samples was used to determine 95% confidence intervals (CIs) to test for statistical significance.

In case of missing data (e.g., due to technical problems), data from this participant for all time points for the affected variable were omitted from analyses. Missing data for each variable are indicated in the result section. All results are reported as mean ± standard error of the mean (SEM) unless indicated otherwise. All authors had access to the study data and reviewed and approved the final manuscript.

## Results

### Participants

Volunteers randomized to practice brief relaxation (*N* = 60) or control (*N* = 60) did not differ with respect to sociodemographic variables or psychosocial questionnaire scores (Table 1, upper section). As per exclusion criteria, mean HADS scores were within the normal range and below the clinically-relevant cut-offs. Nevertheless, mean HADS anxiety score was significantly higher in the control group (*p* = 0.026), and was therefore included as a covariate in subsequent analyses. No significant differences were observed in trait anxiety assessed with the STAI. This is however not unusual given that the HADS measures clinical symptoms of anxiety, while STAI scores primarily reflect non-clinical anxiety. The groups were comparable with respect to all measures collected during the 4-week PMR training phase (Table 1, lower section), including training intensity, frequency, perceived training efficacy, psychological distress, and the CAR (for weekly means, see Table 2). Rectal thresholds, assessed on the study day prior to first randomization, were comparable between groups (sensory threshold: 14.8 ± 0.7 mmHg relaxation group, 15.0 ± 0.7 mmHg control group, *t* = −0.2, *p* = 0.87; pain threshold: 36.6 ± 1.3 mmHg relaxation group, 35.9 ± 1.9 mmHg control group, *t* = −0.5, *p* = 0.65).

**Table 2:** Relaxation training period.

		**Relaxation group**	**Control group**	**Test statistic**	***P***
Mean training time, minutes	Week 1	115.0 (11.7) *n =* 59	97.3 (5.1) *n =* 58	*t =* −1.4	0.17
	Week 2	71.7 (9.2) *n =* 59	61.8 (6.7) *n =* 57	*t =* −0.9	0.39
	Week 3	59.2 (8.8) *n =* 58	49.3 (6.2) *n =* 58	*t =* −0.9	0.36
	Week 4	55.5 (8.3) *n =* 59	55.6 (6.9) *n =* 58	*t =* 0.1	0.99
Mean training units, *N*	Week 1	2.8 (0.2) *n =* 59	2.5 (0.1) *n =* 58	*t =* −1.3	0.20
	Week 2	2.8 (0.2) *n =* 59	2.5 (0.2) *n =* 57	*t =* −1.2	0.24
	Week 3	2.9 (0.2) *n =* 58	2.4 (0.1) *n =* 58	*t =* −2.1	0.04
	Week 4	2.8 (0.2) *n =* 59	2.7 (0.2) *n =* 58	*t =* −0.3	0.73
Perceived training efficacy^a^	Week 1	4.2 (0.2) *n =* 58	4.4 (0.2) *n =* 58	U = −0.4	0.72
	Week 2	4.5 (0.2) *n =* 59	4.7 (0.1) *n =* 57	U = −0.6	0.56
	Week 3	4.7 (0.2) *n =* 59	4.8 (0.2) *n =* 57	U = −0.2	0.84
	Week 4	4.7 (0.2) *n =* 58	4.7 (0.2) *n =* 58	U = −0.1	0.99
Mean distress^b^	Week 1	4.1 (0.1) *n =* 59	4.1 (0.2) *n =* 58	U = −0.2	0.84
	Week 2	4.0 (0.2) *n =* 59	4.1 (0.2) *n =* 56	U = −0.3	0.75
	Week 3	3.8 (0.2) *n =* 58	4.0 (0.2) *n =* 57	U = −1.1	0.28
	Week 4	4.1 (0.2) *n =* 57	4.1 (0.2) *n =* 58	U = −0.1	0.98
Cortisol awakening response^c^	Week 1	219.1 (78.3) *n =* 58	248.7 (80.9) *n =* 57	*t =* 0.3	0.79
	Week 2	179.0 (98.6) *n =* 56	248.0 (96.3) *n =* 57	*t =* 0.5	0.62
	Week 3	237.2 (89.1) *n =* 58	253.6 (67.4) *n =* 57	*t =* 0.1	0.88
	Week 4	296.9 (82.7) *n =* 60	368.6 (77.1) *n =* 56	*t =* 0.6	0.53

*Data shown here extend data shown in Table 1. All data are shown as mean ± standard error of the mean. All N = 120 participants underwent the same home-based relaxation training, and were randomized on the study day to brief relaxation vs. control task. Weekly means were compared between groups with independent samples t-tests or Mann-Whitney U-test as appropriate. To assess changes across weeks, ANOVA and Friedman test were used on data from the whole sample (N = 120). Mean training time showed a significant decrease (F = 76.9, p < 0.001, $\eta_{\text{p}}^{\text{2}}=\;0.40$, ANOVA time effect). No significant changes were observed for mean training units (F = 0.5, p = 0.67, $\eta_{\text{p}}^{\text{2}}\;=\;0.01$), perceived training efficacy (Chi^2^ = 4.2, p = 0.24), mean distress (Chi^2^ = 3.2, p = 0.37), or cortisol awakening response (F = 1.5, p = 0.22, $\eta_{\text{p}}^{\text{2}}$ = 0.01). ^a^Perceived training efficacy during the last week, rated on a seven-point Likert-scale ranging from “training worked not at all” to “training worked perfectly.” ^b^Mean distress rated on a seven-point Likert-scale ranging from “felt completely relaxed” to “felt extremely distressed.” ^c^Cortisol awakening response was calculated as area under the curve (AUC) with respect to increase which corrects for baseline levels. Saliva cortisol was collected immediately after, as well as 30, 45, and 60 min after awakening*.

### Stress Markers

The ANCOVA computed to test effects of the brief relaxation (*N* = 60) vs. control intervention (*N* = 60) on stress markers (see Table 3; for group means per treatment suggestion group, see Table 4) revealed significant group × time interactions for systolic blood pressure (*F* = 9.22, *p* < 0.001, η_p_^2^ = 0.08) and heart rate (*F* = 8.10, *p* < 0.001, η_p_^2^ = 0.07), which decreased significantly in the relaxation but not in the control group. Salivary cortisol and state anxiety showed significant decreases over time (salivary cortisol: *F* = 11.68, *p* < 0.001,η_p_^2^ = 0.09; state anxiety scores: *F* = 9.56, *p* < 0.001, η_p_^2^ = 0.08), however, without evidence of significant group × time interactions (salivary cortisol: *F* = 0.07, *p* = 0.86, η_p_^2^ = 0.01; state anxiety scores: *F* = 0.53, *p* = 0.59, η_p_^2^ = 0.01). No significant effects were observed for diastolic blood pressure.

**Table 3:** Stress parameters.

	**Relaxation group (*N* = 60)**	**Control group (*N* = 60)**	**ANCOVA time effect^a^**	**ANCOVA group effect^a^**	**ANCOVA interaction^a^**
**SYSTOLIC BP (mmHg)**					
BASELINE	122.2 ± 1.6	119.9 ± 1.6	*F =* 2.41, *p =* 0.09	*F =* 0.21, *p =* 0.65	*F =* 9.22, p < 0.001
After treatment suggestion	120.0 ± 1.6	117.1 ± 1.4			
After TEST	117.9 ± 1.6^***^	120.5 ± 1.4			
**DIASTOLIC BP (mmHg)**					
BASELINE	80.7 ± 1.1	78.9 ± 1.2	*F =* 0.13, *p =* 0.88	*F =* 0.16, *p =* 0.69	*F =* 0.40, *p =* 0.67
After treatment suggestion	78.7 ± 1.0	77.9 ± 1.0			
After TEST	78.6 ± 1.1	77.9 ± 1.6			
**HEART RATE (beats/min)**					
BASELINE	67.2 ± 1.4^#^	63.1 ± 1.1^#^	*F =* 0.72, *p =* 0.49	*F =* 1.88, *p =* 0.17	*F =* 8.10, *p* < 0.001
After treatment suggestion	64.4 ± 1.4^**^	63.1 ± 1.1			
After TEST	63.6 ± 1.4^***^	64.2 ± 1.2			
**SALIVARY CORTISOL (nmol/l)**					
BASELINE	12.2 ± 1.2	11.2 ± 0.8	*F =* 11.68, *p* < 0.001	*F =* 0.64, *p =* 0.43	*F =* 0.07, *p =* 0.94
After treatment suggestion	10.2 ± 0.9^***^	9.1 ± 0.5^***^			
After TEST	9.8 ± 0.8^***^	8.7 ± 0.6^***^			
**STAI STATE ANXIETY**					
BASELINE	37.4 ± 1.0	38.1 ± 1.2	*F =* 9.56, *p* < 0.001	*F =* 0.07, *p =* 0.79	*F =* 0.53, *p =* 0.58
After treatment suggestion	35.5 ± 1.2	36.1 ± 1.0			
After TEST	31.5 ± 0.9^***^	33.1 ± 0.9^*^			

*All data are shown as mean ± standard error of the mean, unless indicated otherwise. Stress parameters were repeatedly assessed, i.e., at BASELINE (prior to first randomization to relaxation vs. control intervention), after treatment suggestions, and after the series of distensions (TEST). BP, blood pressure; STAI, Spielberger State-Trait Anxiety Inventory (state version)*.

a *Results of analyses of covariances (ANCOVA) accounting for HADS anxiety scores. Incomplete/missing data: Incomplete STAI-S questionnaires: N = 3 relaxation group, N = 2 control group; technical errors with ECG signal for heart rate: N = 3 relaxation group, N = 6 control group*.

* *p < 0.05*,

** *p < 0.01*,

*** *p < 0.001, results of Bonferroni-corrected paired t-tests comparing means vs. baseline within each experimental group*.

# *p < 0.05, result of post-hoc computed Bonferroni-corrected ANCOVA, comparing relaxation and control group at distinct time points*.

**Table 4:** Stress parameters in all experimental groups.

		**Relaxation group**			**Control group**		
		**Treatment suggestions**			**Treatment suggestions**		
		**Positive (*n =* 20)**	**Neutral (*n =* 20)**	**Negative (*n =* 20)**	**Positive (*n =* 20)**	**Neutral (*n =* 20)**	**Negative (*n =* 20)**
Systolic BP (mmHg)	BASELINE	123.8 ± 2.9	122.6 ± 3.2	120.2 ± 2.5	120.4 ± 2.9	119.9 ± 2.0	119.3 ± 3.4
	After treatment suggestion	121.2 ± 2.6	119.5 ± 3.1	119.4 ± 2.6	117.4 ± 2.9	119.1 ± 2.0	114.9 ± 2.3
	After TEST	119.1 ± 2.9	118.3 ± 2.8	116.5 ± 2.5	119.6 ± 2.9	120.7 ± 2.3	121.4 ± 2.4
Diastolic BP (mmHg)	BASELINE	82.7 ± 1.7	80.1 ± 2.4	79.4 ± 1.6	76.9 ± 2.2	81.6 ± 1.9	78.4 ± 1.8
	After treatment suggestion	79.7 ± 1.6	78.5 ± 2.0	78.0 ± 1.7	77.8 ± 2.1	80.2 ± 1.6	75.9 ± 1.6
	After TEST	78.9 ± 2.0	79.1 ± 2.0	77.9 ± 1.6	74.3 ± 4.1	81.1 ± 1.8	78.2 ± 1.4
Heart rate (beats/min)	BASELINE	66.0 ± 1.8	68.7 ± 3.1	66.8 ± 2.2	62.2 ± 1.9	63.8 ± 2.2	63.3 ± 1.8
	After treatment suggestion	63.6 ± 1.9	66.4 ± 2.9	63.3 ± 2.3	62.3 ± 1.9	63.4 ± 2.1	63.5 ± 1.8
	After TEST	62.7 ± 1.8	65.4 ± 3.4	62.8 ± 1.9	64.2 ± 2.0	62.9 ± 2.6	65.4 ± 1.4
Salivary cortisol (nmol/l)	BASELINE	12.2 ± 1.5	12.2 ± 2.7	12.3 ± 1.8	10.6 ± 1.4	12.8 ± 1.6	10.3 ± 1.2
	After treatment suggestion	10.0 ± 1.3	10.1 ± 1.9	10.5 ± 1.8	8.6 ± 0.9	10.4 ± 1.1	8.4 ± 0.6
	After TEST	9.4 ± 1.1	9.8 ± 1.5	10.1 ± 1.7	7.7 ± 0.8	10.1 ± 1.3	8.2 ± 0.7
STAI State anxiety	BASELINE	36.0 ± 1.9	37.0 ± 1.7	39.3 ± 1.6	38.4 ± 2.4	38.3 ± 2.1	37.6 ± 1.9
	After treatment suggestion	33.4 ± 2.2	32.8 ± 1.5	40.5 ± 1.8	34.6 ± 1.8	34.9 ± 1.5	38.7 ± 2.0
	After TEST	30.8 ± 1.5	31.1 ± 1.2	32.7 ± 2.1	33.4 ± 1.2	31.9 ± 1.2	34.4 ± 1.9

*Data shown here extend data shown in Table 3. All data are shown as mean ± standard error of the mean. BP, Blood pressure; STAI, State Trait Anxiety Inventory*.

### Pain-Related Measures

**Figure 1:**
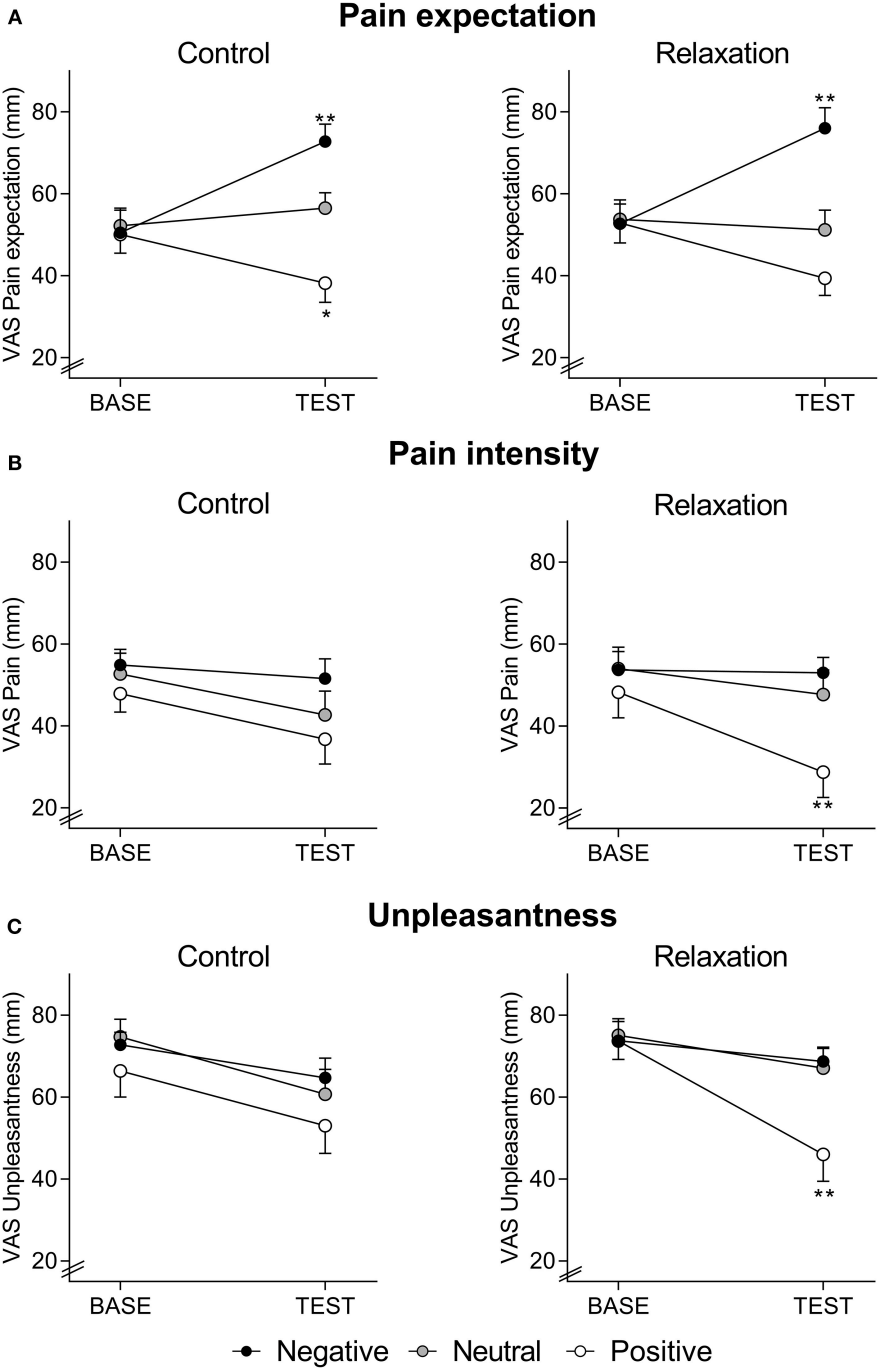
Expected pain intensity **(A)**, perceived pain intensity **(B)**, and perceived pain unpleasantness **(C)**, assessed with visual analog scale (VAS, 0–100 mm) at BASELINE and TEST, in groups receiving positive, neutral, or negative treatment information after relaxation (right panels) or control (left panels). Note that pain expectation was assessed before, whereas perceived pain intensity and unpleasantness were assessed after the series of distensions during BASELINE and TEST, respectively. For ANCOVA results, please see text. *p < 0.05; **p < 0.01 results of planned comparisons with Bonferroni-correction at TEST (for exact *p*-values, see text) comparing groups with positive information to groups with neutral information (to test for placebo effects) and groups with negative information to groups with neutral information (to test for nocebo effects) after either relaxation or control.

Expected pain intensity (Figure 1A) was reduced by positive and increased by negative treatment suggestions (*F* = 8.84, *p* < 0.001, η_p_^2^ = 0.14, ANCOVA main effect of treatment information; *F* = 32.25, *p* < 0.001, η_p_^2^ = 0.37, ANCOVA interaction effect of time × treatment information). Pain expectation was not affected by relaxation, as indicated by the absence of significant main or interaction effects.

For perceived pain intensity (Figure 1B), there was a significant effect of treatment suggestions (*F* = 4.38, *p* = 0.015 η_p_^2^ = 0.07, time x suggestion interaction; *F* = 3.70, *p* = 0.028 η_p_^2^ = 0.06, main effect of suggestion), but no main effect of the intervention (*F* = 0.01, *p* = 0.98 η_p_^2^ = 0.01, time x intervention interaction; *F* = 0.31, *p* = 0.58 η_p_^2^ = 0.01, main effect of intervention) and no interaction effect (*F* = 1.29, *p* = 0.29 η_p_^2^ = 0.02, time × suggestion × intervention interaction). Planned comparisons of group means revealed significantly reduced perceived pain intensity at TEST due to positive compared to neutral suggestions in the relaxation groups (*F* = 8.04, *p* = 0.008, η_p_^2^ = 0.19), while a similar placebo effect was not observed in the control groups (*F* = 0.44, *p* = 0.51, η_p_^2^ = 0.01). Nocebo effects, tested by comparing groups with negative vs. neutral treatment suggestions, were not observed in either intervention group (relaxation: *F* = 0.3, *p* = 0.57, η_p_^2^ = 0.01; control: *F* = 1.9, *p* = 0.17, η_p_^2^ = 0.05).

For pain unpleasantness (Figure 1C), a significant interaction between intervention, treatment suggestions, and time (*F* = 3.53, *p* = 0.032, η_p_^2^ = 0.06), as well as a significant effect of treatment suggestions (*F* = 4.41, *p* = 0.014 η_p_^2^ = 0.07, time × suggestion interaction; *F* = 3.21, *p* = 0.044 η_p_^2^ = 0.05, main effect of treatment suggestion) emerged, while effects of the intervention were not significant (*F* = 0.82, *p* = 0.37, η_p_^2^ = 0.01, time × intervention interaction; *F* = 0.37, *p* = 0.54 η_p_^2^ = 0.01, main effect of intervention). Planned comparisons of group means revealed significantly reduced unpleasantness at TEST in response to positive when compared to neutral suggestions (*F* = 7.8, *p* = 0.008, η_p_^2^ = 0.18) in relaxation groups, but not in control groups (*F* = 0.9, *p* = 0.34, η_p_^2^ = 0.02). No significant effects of negative suggestions were observed (relaxation groups: *F* = 0.02, *p* = 0.88, η_p_^2^ = 0.01; control groups: *F* = 0.63, *p* = 0.43, η_p_^2^ = 0.02).

### Exploratory Correlational and Mediation Analyses

To explore the role of pain expectation, we conducted correlational and mediation analyses both in the whole sample and in groups with positive suggestions (placebo groups) and negative suggestions (nocebo groups). In the whole sample of *N* = 120, pain expectation was significantly associated with both perceived pain intensity (*r* = 0.58, *p* < 0.001) and pain unpleasantness (*r* = 0.38, *p* < 0.001). In addition, pain expectation correlated with state anxiety (*r* = 0.25, *p* = 0.007), but not with other stress markers. No significant correlations between any other stress marker and pain outcomes were found (all *p* > 0.05, data not shown).

**Figure 2:**
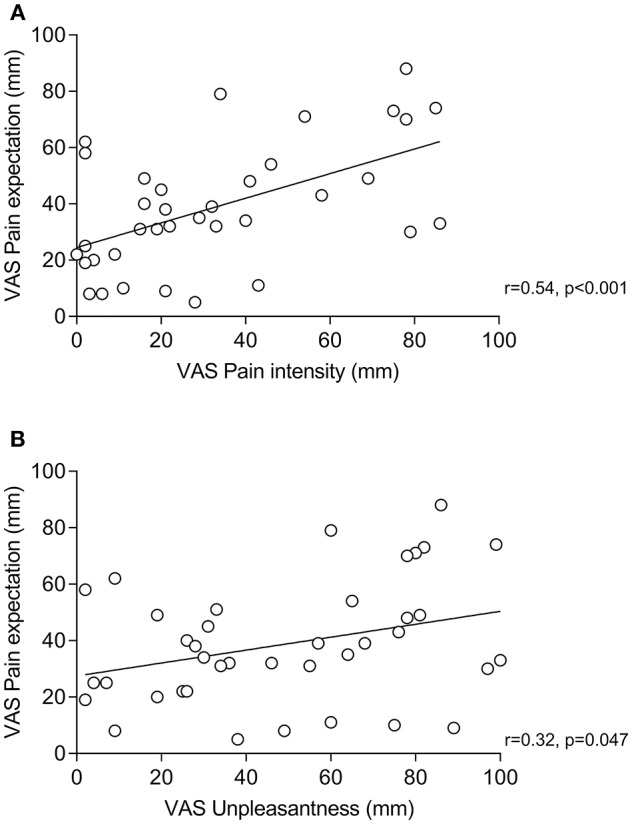
Correlations (Pearson's r) between pain expectation and perceived pain intensity **(A)** and pain expectation and perceived pain unpleasantness **(B)** within groups receiving positive treatment suggestions (i.e., placebo groups).

**Figure 3:**
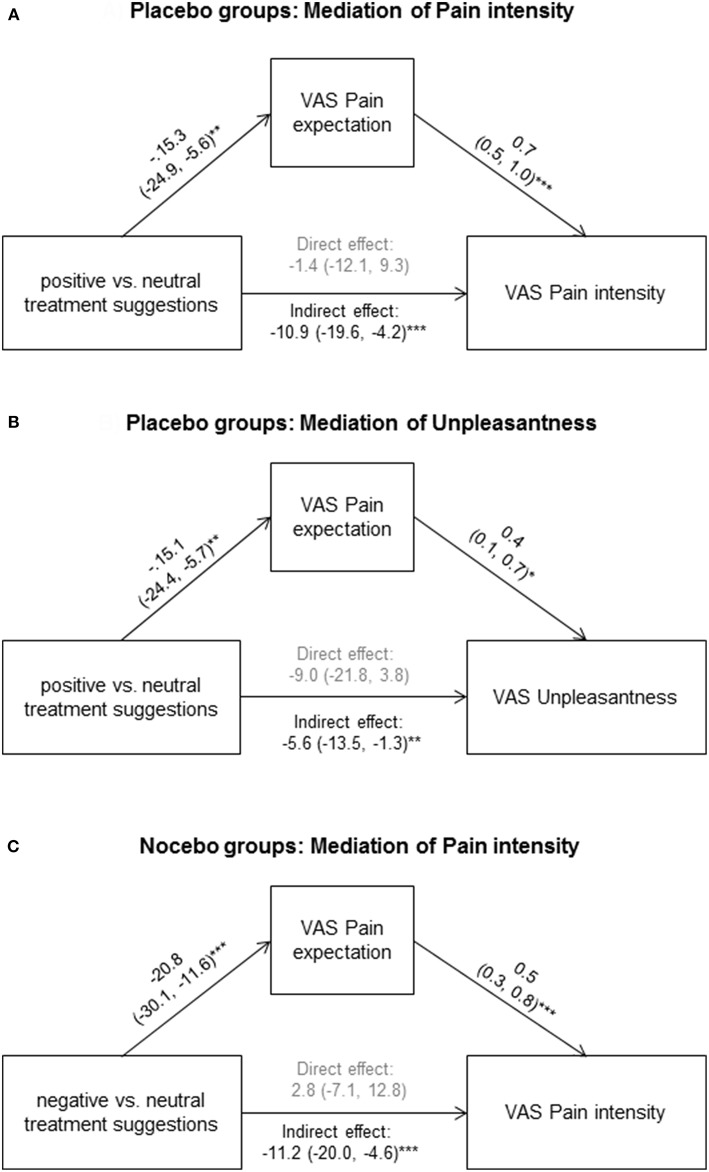
To explore if pain expectation mediated effects of positive treatment suggestions, we conducted mediation analyses on data from placebo and neutral suggestion groups for pain intensity **(A)** and unpleasantness **(B)**, as well as on data from nocebo and neutral suggestion groups for pain intensity **(C)**. Standardized coefficients with 95% CIs are shown. *p < 0.05; **p < 0.01; ***p < 0.001.

Within placebo groups (*N* = 40), pain expectation was positively correlated with perceived pain intensity (*r* = 0.54, *p* < 0.001, Figure 2A) and unpleasantness (*r* = 0.32, *p* = 0.047, Figure 2B). To explore if pain expectation mediated effects of positive treatment suggestions, we conducted mediation analyses on data from placebo and neutral suggestion groups (*N* = 80) after ensuring that positive associations remained significant in multiple regression analyses including treatment suggestions in addition to pain expectation as independent variables (data not shown). We found an indirect effect of pain expectation which mediated the association between treatment suggestions and pain intensity (Figure 3A) as well as unpleasantness (Figure 3B).

Within nocebo groups (*N* = 40), pain expectation was significantly associated pain intensity (*r* = 0.53, *p* = 0.03), but not with unpleasantness (*r* = 0.25, *p* = 0.11). The former association remained significant in multiple regression analyses including treatment suggestions in addition to pain expectation as independent variables (data not shown). To explore if pain expectation mediated effects of negative treatment suggestions, we conducted mediation analysis for pain intensity on data from nocebo and neutral suggestion groups (*N* = 80). We found an indirect effect of pain expectation which mediated the association between treatment suggestions and pain intensity (Figure 3C).

We conducted additional mediation analyses to explore if putative effects of relaxation vs. control on pain intensity or unpleasantness could be explained by pain expectation. In separate analyses within the placebo, nocebo, and control groups, we did not find evidence of direct or indirect effects of relaxation on pain outcomes (data not shown). This is in line with (1) the absence of significant effects of relaxation on expectations and (2) the non-significant correlations between stress markers and outcome variables.

## Discussion

This is the first study testing whether a behavioral intervention aimed at reducing acute stress parameters affects the response to positive and/or negative treatment suggestions in a clinically-relevant model of visceral pain. Our findings suggest that a brief relaxation exercise may facilitate the induction of placebo analgesia by positive when compared to neutral treatment suggestions. These findings extend evidence that placebo analgesia can be boosted with pharmacological interventions (19, 20). There are clearly many facets surrounding the psychosocial treatment context that ultimately determine the presence and magnitude of expectancy effects. Our results support the contribution of relaxation and stress as psychobiological states within the psychosocial treatment context—factors which clearly deserve more attention in translational studies aiming to maximize positive expectancy effects in clinical settings.

Healthy volunteers were randomized to a brief muscle relaxation exercise or a control task just prior to randomly receiving deceptive positive, deceptive negative, or truthful neutral treatment suggestions regarding an intravenous infusion that was in reality saline in all groups. These treatment suggestions induced group-specific changes in pain expectation, with *reduced* pain expectation in groups receiving positive suggestions of pain relief (i.e., placebo groups) and *increased* pain expectation in groups receiving negative suggestions of enhanced pain sensitivity (i.e., nocebo groups). While the relaxation exercise had no discernable effect on pain expectation, relaxation significantly interacted with positive treatment suggestions. Planned comparisons of group means showed significantly reduced pain intensity and lower pain unpleasantness after positive compared to neutral treatment suggestions only in the relaxation groups. In other words, positive treatment suggestions only induced placebo analgesia in relaxed participants, which is partly in line with our hypothesis assuming a facilitated placebo effect. On the other hand, relaxation had no discernable effect on groups receiving negative suggestions. Since no nocebo effects were observed in either relaxation or control group, we could not confirm our hypothesis that relaxation may reduce nocebo hyperalgesia.

We chose a brief relaxation exercise as behavioral intervention with the intention to acutely reduce stress parameters within a highly standardized psychosocial treatment context. This approach was conceptually and methodologically based on our earlier brain imaging work on the role of emotional context in visceral pain processing (38). It complements placebo/nocebo studies in the broader pain field aiming to discern effects of acute stress, state anxiety or fear (23, 25, 26, 28–30, 40) on placebo analgesia or nocebo hyperalgesia. In order to verify the efficacy of the intervention and to gain insight into possible mechanisms, we assessed several relevant stress markers reflecting different biopsychological aspects of stress. Brief relaxation significantly reduced systolic blood pressure and heart rate, supporting effects on the autonomic nervous system (ANS). On the other hand, no effects on state anxiety or cortisol concentrations were found. This could indicate that measures of ANS function (herein: heart rate and blood pressure) are more sensitive or responsive to short-term effects of PMR, at least in healthy individuals. However, it should be noted that cortisol and state anxiety significantly decreased in both groups, and that these measures could not be assessed immediately after the relaxation exercise for methodological considerations. Hence, effects on state anxiety or cortisol could be difficult to detect given reductions in both groups and may have been missed herein. Nevertheless, the ANS is increasingly appreciated in the context of pain modulation [e.g., (41)], especially in acute and chronic visceral pain as a key component of the brain axis (42–50). Within the placebo field, the ANS has been proposed as a primary mediator of peripheral placebo effects in conditioning models (51, 52). Placebo analgesia evokes complex effects within the cardiovascular system, including changes in heart rate and blood pressure (25, 53). Blood pressure and stress were found to mediate hyperalgesia after nocebo suggestions (27), and a recent study supports a role of autonomic arousal in the persistence of nocebo hyperalgesia (54). Interestingly, the same study (54) found no correlation between either self-reported anxiety or autonomic arousal and placebo analgesia/nocebo hyperalgesia. We also explored these relationships in our dataset, and found no correlations between placebo effects and stress markers. In fact, pain expectation was the only mediator we could identify to explain the association between treatment suggestions and pain-related outcomes. These results call for caution with respect to any speculation about stress-related mechanisms and underscore the need to further study possible moderators of placebo analgesia, especially emotional factors that have been proposed to play a role in placebo analgesia (55, 56). Clearly, visceral pain modulation is complex and involves many cognitive, emotional, and possibly neurobiological factors that remain to be fully understood.

This study has strengths and limitations. Strengths include the clinically-relevant visceral pain model, blinding procedures, the combination of different psychobiological measures for traits and states, and the inclusion of groups receiving positive, negative or neutral treatment suggestions within one study. The full factorial within-between study design goes beyond correlational approaches aiming to identify psychological mediators and moderators of placebo and nocebo effects. At the same time, final group *N*s are relatively small, posing limitations of statistical power, and risk of Type II error. This may for example explain why *post hoc* testing revealed a statistically significant reduction in pain expectation induced by positive vs. neutral suggestions only in the control but not in the relaxation group. Further, for reasons of feasibility and cost effectiveness, data from the control group were also used in Roderigo et al. (30), and there was also no additional control group that did not undergo prior relaxation training for feasibility reasons and to ensure blinding and randomization on the study day. We therefore cannot assess possible effects of prior relaxation training on measures obtained on the study day. While the absence of the brief PMR vs. control exercise effects on pain-related outcomes on the study day may be interpreted as evidence supporting a lack of relaxation effects on visceral pain, this would in our view be premature. First, we could not ascertain whether regular PMR exercise of 4 weeks did in fact induce changes in variables relevant to chronic stress. To do so was not our intention since this was not a treatment study but rather herein implemented in order to teach a sufficiently large number of study volunteers to perform PMR on the study day, aiming to realize a study design with proper randomization and blinding. We recruited a tightly-screened, healthy population of young individuals with comparatively low levels of chronic stress or stress-related symptom burden. Hence, our findings likely do not transfer to other populations at risk for stress-related health conditions or even patients with chronic pain, and should not be viewed as evidence for or against the potential clinical use of relaxation techniques in patients. In irritable bowel syndrome, for example, a recent meta-analysis (57) showed a clinical benefit of relaxation methods, and an older, more comprehensive Cochrane review (58) on relaxation therapy and stress management revealed medium effect sizes for symptom severity after 2–3 months, but inconsistent longer-term findings (after 6–12) months with regard to abdominal pain and quality of life. The lack of control group without prior relaxation training further limits our ability to test the possibility that the absence of nocebo effects could be explained by effect(s) of previous relaxation training. There are other methodological considerations regarding the absence of nocebo effects herein: Given clear effects of negative suggestions on expected pain intensity, we would argue that the nocebo manipulation did not “fail” *per se*. This is supported by positive correlations between pain expectation and intensity and to a smaller extent pain with unpleasantness, supporting the connection between negative pain-related expectations and ratings. Whether, negative expectations are more tightly “linked” with intensity than unpleasantness requires further study. Nocebo effects in visceral pain models have thus far not been studied outside of our group, and they may be more difficult to reliably elicit in the laboratory setting than placebo effects. It is conceivable that they can more effectively be induced in healthy individuals under conditions of heightened stress or arousal, e.g., in the scanner setting (33) that is *per se* stressful (59) or after acute psychosocial stress as shown in a separate arm of this study (30). Our nocebo paradigm relied exclusively on treatment suggestions, and the study was only powered to detect large effects. Combining suggestions with a learning experience (i.e., a preconditioning procedure consisting of the surreptitious increase/decrease of pain intensity prior to suggestions) may be more efficacious and enhance effect size (13, 22). Finally, our approach to utilize truthful information regarding i.v. administration of saline as a control (i.e., groups with “neutral suggestions”) is essential to properly quantify placebo/nocebo effects and distinguish them from other effects, like habituation, sensitization, order effects, etc. At the same time, these “neutral” groups are not untreated and hence by definition not free of treatment-related expectations. This may also reduce the magnitude of expectancy effects when their detection essentially relies on group comparisons [for more detailed methodological considerations, see (13)].

Together, our data provide further evidence that psychological states may alter how individuals respond to treatment suggestions. They complement recent conceptual developments on how bodily symptoms are experienced (60), especially interoceptive symptoms (61) which are demonstrably particularly salient and unpleasant when compared to exteroceptive, somatic stimuli even at matched intensities (62). Inter-individual variability in the presence and magnitude of placebo and nocebo effects is likely not only moderated by individual traits, characteristics of the treatment, and patient-provider interactions, but also by the psychological state in which treatment expectations are formed. Our findings call for more research to unravel how psychological states and their neurobiological correlates contribute to inter-individual variability in expectancy effects on symptom perception. Further, these experimental data acquired in a clinically-relevant pain model pave the way toward translation into clinical populations implementing behavioral interventions that target patients' expectancies and (also) consider psychobiological states. Indeed, placebo and nocebo effects for interoceptive, visceral symptoms are relevant to the treatment of the large group of patients with functional gastrointestinal disorders like IBS (12), but studies are needed to test whether findings from healthy volunteers can be transferred to patients. The role of the psychobiological stress systems in the pathophysiology of these clinical conditions is undisputable, as is the importance of pain or symptom-related cognitive and emotional factors (12, 42, 63, 64). If indeed these very same systems (or one of these) impacts how treatment expectations are processed, the implications are broad both for clinical practice and treatment trials. Indeed, placebo research has impressively demonstrated the clinical potential offered by psychological interventions (1, 2, 11), especially in the context of pain (1, 4). Effort to transfer knowledge from mechanistic work to clinical routine (65) are built on evidence that placebo analgesia engages similar neurobiological mechanisms as those responsible for the efficacy of pharmacological analgesic treatment (11, 66), and effectively enhances the “pure” pharmacological effect of analgesics in experimental but also in clinical settings (1, 2, 4, 18). Together, these findings pave the way for future studies. Our findings provide a small, additional “piece of the puzzle,” at minimum supporting that the recent statement “Implementation of successful treatment requires effective communication skills to improve patient acceptance, adherence and to optimize the patient provider relationship.” (67) may need amendment to incorporate additional aspects of the psychosocial treatment context, including individual treatment expectations and psychobiological states.

## Data Availability

The raw data supporting the conclusions of this manuscript will be made available by the authors, without undue reservation, to any qualified researcher.

## Author Contributions

SB and SE: planning of the study and acquisition of funding. TR and SB: conducting the study. TR, SB, SE, and PE: data analysis and interpretation. SE, SB, and PE: drafting of the manuscript. All authors: revision of the manuscript for critical intellectual content and approval of the final draft submitted. All authors agree to be accountable for all aspects of the work in ensuring that questions related to the accuracy or integrity of any part of the work are appropriately investigated and resolved.

### Conflict of Interest Statement

The authors declare that the research was conducted in the absence of any commercial or financial relationships that could be construed as a potential conflict of interest.
